# Biological event composition

**DOI:** 10.1186/1471-2105-13-S11-S7

**Published:** 2012-06-26

**Authors:** Halil Kilicoglu, Sabine Bergler

**Affiliations:** 1 Department of Computer Science and Software Engineering, Concordia University, 1455 de Maisonneuve Blvd. West, Montréal, Canada

## Abstract

**Background:**

In recent years, biological event extraction has emerged as a key natural language processing task, aiming to address the information overload problem in accessing the molecular biology literature. The BioNLP shared task competitions have contributed to this recent interest considerably. The first competition (BioNLP'09) focused on extracting biological events from Medline abstracts from a narrow domain, while the theme of the latest competition (BioNLP-ST'11) was *generalization *and a wider range of text types, event types, and subject domains were considered. We view event extraction as a building block in larger discourse interpretation and propose a two-phase, linguistically-grounded, rule-based methodology. In the first phase, a general, underspecified semantic interpretation is composed from syntactic dependency relations in a bottom-up manner. The notion of *embedding *underpins this phase and it is informed by a trigger dictionary and argument identification rules. Coreference resolution is also performed at this step, allowing extraction of inter-sentential relations. The second phase is concerned with constraining the resulting semantic interpretation by shared task specifications. We evaluated our general methodology on core biological event extraction and speculation/negation tasks in three main tracks of BioNLP-ST'11 (GENIA, EPI, and ID).

**Results:**

We achieved competitive results in GENIA and ID tracks, while our results in the EPI track leave room for improvement. One notable feature of our system is that its performance across abstracts and articles bodies is stable. Coreference resolution results in minor improvement in system performance. Due to our interest in discourse-level elements, such as speculation/negation and coreference, we provide a more detailed analysis of our system performance in these subtasks.

**Conclusions:**

The results demonstrate the viability of a robust, linguistically-oriented methodology, which clearly distinguishes general semantic interpretation from shared task specific aspects, for biological event extraction. Our error analysis pinpoints some shortcomings, which we plan to address in future work within our incremental system development methodology.

## Background

The overwhelming amount of new knowledge in molecular biology and its exponential growth necessitate sophisticated approaches to managing molecular biology literature. By providing efficient access to the relevant literature, such approaches are expected to assist researchers in generating new hypotheses and, ultimately, new knowledge. Natural language processing (NLP) techniques increasingly support advanced knowledge management and discovery systems in the biomedical domain [1,2]. In biomedical NLP, biological event extraction is one task that has been attracting great interest recently, largely due to the availability of the GENIA event corpus [3] and the resulting shared task competition (BioNLP'09 Shared Task on Event Extraction [4]). In addition to systems participating in the shared task competition [4], several studies based on the shared task corpus have been reported [5-7], the top shared task system has been applied to PubMed scale [1], and biological corpora targeted for event extraction in other biological subdomains have been constructed [8]. Furthermore, UCompare, a meta-service providing access to some of the shared task systems [9], have been made available.

One of the criticisms towards the corpus annotation/competition paradigm in biomedical NLP has been that they are concerned with narrow domains and specific representations, and that they may not generalize well. The GENIA event corpus, for instance, contains only Medline abstracts on transcription factors in human blood cells. Whether models trained on this corpus would perform well on full-text articles or on text focusing on other aspects of biomedicine (e.g., treatment or etiology of disease) remains largely unclear. Since annotated corpora are not available for every conceivable subdomain of biomedicine, it is desirable for automatic event extraction systems to be generally applicable to different types of text and domains without requiring much training data or customization.

In the follow-up event to BioNLP'09 Shared Task on Event Extraction, organizers of the second shared task (BioNLP-ST'11) [10] address this criticism to some extent. The theme of BioNLP-ST'11 is *generalization *and the net is cast much wider. There are 4 event extraction tracks: in addition to the GENIA track that again focuses on transcription factors [10], the epigenetics and post-translational modification track (EPI) focuses on events relating to epigenetic change, such as DNA methylation and histone modification, as well as other common post-translational protein modifications [11], whereas the infectious diseases track (ID) focuses on bio-molecular mechanisms of infectious diseases [11]. Both GENIA and ID tracks include data from full-text articles in addition to abstracts. Detection of event modifications (speculation and negation) is an optional task in all three tracks. The fourth track, Bacteria [12], consists of two sub-tracks: Biotopes (BB) and Interactions (BI). We provide a summary of the BioNLP-ST'11 tracks in 1.

**Table 1:** An overview of BioNLP-ST'11 tracks

	GENIA	EPI	ID	BB	BI
Number of core events	9	15	10	2	10

Triggers annotated?	Y	Y	Y	N	N

Includes full-text?	Y	N	Y	N	N

Speculation/Negation?	Y	Y	Y	N	N

An overview of BioNLP-ST'11 tracks.

BioNLP-ST'11 provides a good platform to validate some aspects of our general research, in which we are working towards a linguistically-grounded, bottom-up semantic interpretation scheme. In particular, we focus on lower level discourse phenomena, such as *modality*, *negation*, and *causation *and investigate how they interact with each other, as well as their effect on basic propositional semantic content (who did what to who?) and higher discourse/pragmatics structure. We subsume these phenomena of study under the notion of *embedding*. In our model, we distinguish two layers of predications: *atomic *and *embedding*. An *atomic predication *corresponds to the elementary unit and lowest level of relational meaning: in other words, a semantic relation whose arguments correspond to ontologically simple entities. Atomic predications form the basis for *embedding predications*, that is, predications taking as arguments other predications. We hypothesize that the semantics of the embedding layer is largely domain-independent and that treating this layer in a unified manner can benefit a number of natural language processing tasks, including event extraction and speculation/negation detection.

We participated in three BioNLP-ST'11 tracks: GENIA, EPI, and ID. In the spirit of the competition, we aimed to demonstrate a generalizable methodology that separated domain-independent linguistic aspects from task-specific concerns and that required little, if any, customization or training for individual tracks. Towards this goal, we use a two-phase approach. The first phase (*Composition*) is an implementation of the bottom-up semantic interpretation scheme mentioned above. It takes the concerns of general English into account and is intended to be fully general. It is syntax-driven, presupposes simple entities, a trigger dictionary and syntactic dependency relations, and creates a partial semantic representation of the text. Addressing coreference resolution to some extent at this phase, we also aim to move to the inter-sentential level. Our overall structural approach in the composition phase is in the tradition of graph-based semantic representations [13] and its output bears similarities to the deep-syntactic level of representation proposed in the Meaning-Text Theory [14]. In the second phase (*Mapping*), we rely on shared task event specifications to map relevant parts of this semantic representation to event annotations. This phase is more domain-specific, although the kind of domain-specific knowledge it requires is largely limited to event specifications and event trigger expressions. In addition to extracting core biological events, our system also addresses speculation and negation detection within the same framework. We achieved competitive results in the shared task competition, demonstrating the feasibility of a general, rule-based methodology while avoiding low recall, often associated with rule-based systems, to a large extent. In this article, we extend the work reported in our previous shared task article [15], by integrating coreference resolution into the system, providing a more extensive and formal description of the framework and extending the error analysis.

## Methodology

**Figure 1:**
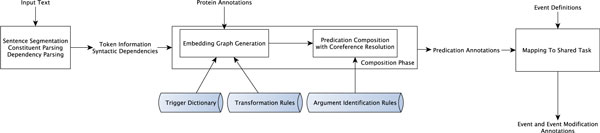
**The shared task pipeline**. The biological event composition pipeline. The cylindrical boxes represent the resources used.

In this section, we first define *atomic *and *embedding *predications and illustrate them using examples from the shared task corpus. Next, we describe a semantic categorization of embedding types, which underpins the creation of embedding predications. After discussing the construction of the trigger dictionary, we present our two-phase approach: the *composition *phase, informed by the trigger dictionary and syntactic dependency relations, and the *mapping *phase, informed by shared task constraints. Finally, we discuss coreference resolution in our framework, a subtask in the composition phase. The shared task pipeline is graphically illustrated in Figure 1.

### Atomic vs. embedding predications

**Definition 1**. A **predication **Pr is an n-ary abstract semantic object that consists of a predicate P and n arguments.

$$Pr:=\left[P,Arg_{1..n}\right]$$

**Definition 2**. A semantic object T is **ontologically simple **if it takes no arguments. A predication takes arguments and is an **ontologically complex **object.

**Definition 3**. A predication is **atomic**, if all of its arguments are ontologically simple.

$$Pr_{atomic}:=\left[P,T_{1..n}\right]$$

**Definition 4**. A predication is **embedding**, if it has at least one ontologically complex argument.

$$Pr_{embedding}:=\left[P,Arg_{\text{1}..n}\right],\text{ where }\left(\exists Arg_{i}:Arg_{i}\in PR\right)\text{ and}\;PR\;\text{is the set of all predications}.$$

**Definition 5**. A **surface element ***SU *is a single token or a contiguous multi-token unit, which may be associated with an abstract semantic object SEM.

• A surface item that is associated with a semantic object is said to be **semantically bound **(⟦*SU*⟧ = SEM).

• Otherwise, it is said to be **semantically free **(⟦*SU*⟧ = ∅).

**Figure 2:**
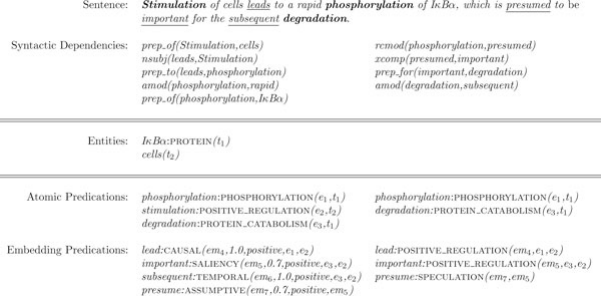
**Atomic vs. embedding predications**. Atomic and embedding predications extracted from the sentence *Stimulation of cells leads to a rapid phosphorylation of IκBα, which is presumed to be important for the subsequent degradation*. from the Medline abstract 7499266. The middle column in the predication rows shows the predications after the *Composition *phase and the right column the event and event modification annotations after the *Mapping *phase. Note that *e*_2 _is not mapped to an event annotation, since its argument is semantically empty, whereas *em*_6 _is not mapped since its semantic type, TEMPORAL, is not relevant in the shared task context. The relevant syntactic dependency relations as well as the entities are illustrated.

Consider the sentence in Figure 2, taken from the Medline abstract with PMID 7499266. Ontologically simple entities, atomic and embedding predications are illustrated. For example, the surface element *IκBα *corresponds to an ontologically simple entity, whose semantic type is PROTEIN. The surface item *cells *is semantically free. As illustrated in Figure 2, we denote ontologically simple entities as *m:*SEM*(id)*, where *m *corresponds to the textual mention of the entity, SEM to its semantic type, and *id *to its unique identifier. We treat semantically free elements as ontologically simple entities, whose semantic types are not known, and represent them as *m(id)*.

Atomic predications in the same sentence are indicated with the identifiers *e*_1_, *e*_2_, and *e*_3 _in Figure 2. The predicates that trigger the atomic predications in the sentence are shown in bold. At the syntactic level, atomic predications prototypically correspond to verbal and nominalized predicates and their syntactic arguments. We denote atomic predications as *m:*SEM*(id,t*_1..*n*_*)*, where *m *corresponds to the predicate mention and *t*_1..*n *_refer to ontologically simple argument(s) of the atomic predication. SEM is the semantic type of the predicate, and by extension, of the predication. Semantic types of atomic predications are event types from the shared task specifications, where applicable.

Underlined expressions in the sentence (*leads*, *presumed*, *important*, and *subsequent*) trigger embedding predications (*em*_4..7_) and indicate higher level information relating biological processes indicated by atomic predications: *leads*, *important *and *subsequent *are used to make *causal *and *temporal *connections between these processes and *presumed *to indicate an *assumption*, though seemingly unproven, towards one of these connections. Syntactically, in addition to verbal and nominalized predicates and their syntactic arguments, embedding predications are also realized via subordination, complementation, and various syntactic modifications. For example, in the example in Figure 2, *em*_6 _is triggered by adjectival modification and *em*_7 _by infinitival complementation.

In the shared task setting, embedding predications correspond to complex regulatory events (e.g., POSITIVE_REGULATION, CATALYSIS) as well as event modifications (NEGATION and SPECULATION), whereas atomic predications largely correspond to simple event types (e.g., GENE_EXPRESSION, PHOSPHORYLATION).

With respect to representation of embedding predications in Figure 2, two points are noteworthy: (a) the semantic types (e.g., CAUSAL, TEMPORAL) are taken from an embedding categorization scheme, and (b) the embedding predications include two new elements: a *scalar modality value *in the [0, 1] range (MV), and a *polarity value *(POL). We revise the predication definition (Definition 1) here to include these elements.

$$Pr:=\left[P,MV,POL,Arg_{1..n}\right]$$

In this paper, MV and POL values will be omitted from representation of atomic predications when they are not relevant to the discussion. We describe the embedding categorization scheme, as well as the modality value and polarity elements in more detail in the next section.

Note that the level of embedding in a sentence can be arbitrarily deep. For example, *em*_7 _takes as argument another embedding predication, *em*_5_, which, in turn, takes atomic predications *e*_2 _and *e*_3 _as arguments.

**Definition 6**. A predication Pr_1 _**embeds **a predication Pr_2 _if Pr_2 _is an argument of Pr_1_.

$$Pr_{1}=\left[P_{1},..Pr_{2},..\right]$$

**Definition 7**. A predication Pr_2 _is within the **scope **of a predication Pr_1 _(written as Pr_1 _*>*Pr_2_), if one of the following conditions is met:

• Pr_1 _embeds Pr_2_.

• There is a predication Pr_3_, such that Pr_1 _embeds Pr_3 _and Pr_2 _is within the scope of Pr_3_.

$$\left(Pr_{1}=\left[P_{1},..Pr_{2},..\right]\right)\vee \left(\left(Pr_{3}>Pr_{2}\right)\wedge Pr_{1}=\left[P_{1},..Pr_{3},..\right]\right)\Rightarrow Pr_{1}>Pr_{2}$$

In the example sentence in Figure 2, the atomic predications *e*_2 _and *e*_3 _are within the scope of *em*_5 _and *em*_7_, and by the same token, *em*_7 _embeds *em*_5_, which embeds *em*_2 _and *em*_3_:

$$em_{7}>em_{5}>\left\{e_{2},e_{3}\right\}$$

Incorporating entity annotations provided by the shared task organizers (ontologically simple, semantically bound entities of PROTEIN type), the first phase of our system (*composition*) is essentially concerned with compositionally building predications, illustrated in the first column of Figure 2. The second phase, *mapping*, deals with converting and filtering these predications to obtain the shared task-specific annotations in the second column of Figure 2.

### Embedding categorization

**Figure 3:**
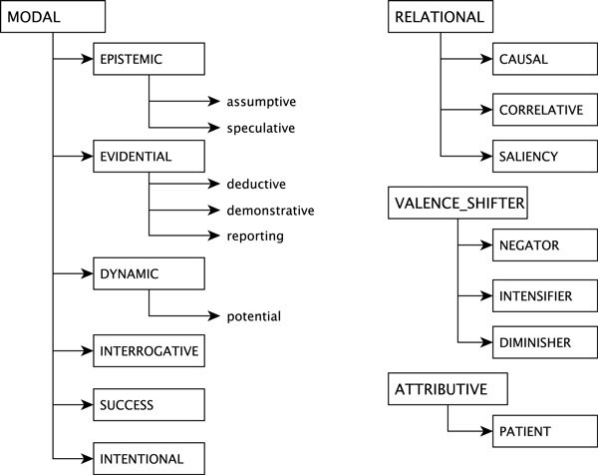
**Embedding predication categorization**. Embedding predication categorization relevant to the shared task.

Our goal in categorizing embedding types is to pinpoint the kind of semantic information indicated with such predicates and to explore their interactions. We draw from existing linguistic typologies and classifications, where appropriate. We distinguish four basic classes of embedding predicates: MODAL, ATTRIBUTIVE, RELATIONAL and VALENCE_SHIFTER. We provide a brief summary below and present the portion of the classification that pertains to the shared task in Figure 3.

#### MODAL type

**Definition 8**. A **modal **predicate modifies the status of the embedded predication with respect to a modal scale (e.g., certainty, possibility, necessity).

Four generally accepted types of modal predicates are given below (cf. [16]), and they are illustrated with sentences from the shared task corpus. The embedded predicate is in bold, and the modal predicate is underlined.

**EPISTEMIC** indicates *judgement *about the status of the embedded predication, and affects its factuality. Subtypes include ASSUMPTIVE and SPECULATIVE.

(1)   (a) *... phosphorylation of IκBα, which is presumed to be **important **for the subsequent degradation*.

(b) *presume:*ASSUMPTIVE(*em*_1_*,0.7,positive,em*_2_) ^ *important*(*em*_2_*...*)

**EVIDENTIAL **indicates *evidence *surrounding the predication, indirectly affecting its factuality according to the evidence source and reliability. Subtypes are DEDUCTIVE, DEMONSTRATIVE, and REPORTING.

(2)   (a) *Our previous results show that recombinant gp41 ... **stimulates **interleukin-10 (IL-10) production ...*

(b) *show:*DEMONSTRATIVE(*em*_1_*,1,positive,em*_2_*,t*_1_) ^ *our-previous-results(t*_1_*) *^ *stimulate(em*_2_*...)*

**DYNAMIC **indicates *ability *or *willingness *of an agent towards an event, corresponding to POTENTIAL and VOLITIVE categories, respectively.

(3)   (a) *Other unidentified ETS-like factors ... are also capable of **binding **GM5*.

(b) *capable:*POTENTIAL(*em*_1_*,1,positive,e*_2_*) *^ *bind(e*_2_*...)*

**DEONTIC **indicates *obligation *or *permission *from an external authority for an event, corresponding to OBLIGATIVE and PERMISSIVE categories, respectively.

(4)   (a) *... future research in this area should be **directed **toward the understanding ...*

(b) *should:*OBLIGATIVE(*em*_1_*,0.7,positive,e*_2_*) *^ *direct(e*_2_*...)*

We consider three additional modal types: INTENTIONAL, INTERROGATIVE, SUCCESS. These types are mentioned in discussions of modality and are sometimes adopted as separate categories; however, there appears to be no firm consensus on their modal status. We chose to include them in our categorization, since corpus analysis provides clear evidence that they affect the status of predications they embed and that they occur in considerable amounts.

**INTENTIONAL **indicates *effort *of an agent to perform an event (cf. [17,18]).

(5)   (a) *... we tried to **identify **downstream target genes regulated by TAL1 ...*

(b) *try:*INTENTIONAL(*em*_1_*,1,positive,e*_2_) ^ *identify(e*_2_*...)*

**INTERROGATIVE **indicates *questioning *of the predication (cf. [19,20]).

(6)   (a) *... we examined whether ... IL-10 up-regulation is **mediated **by the ... synergistic activation of cAMP and NF-κB pathways*.

(b) *examine:*INTERROGATIVE(*em*_1_*,1,positive,em*_2_) ^ *mediate(em*_2_*...)*

**SUCCESS **indicates the *degree of success *associated with the predication (cf. [18,21]).

(7)   (a) *In contrast, gp41 failed to **stimulate **NF-κB binding activity ... *(SUCCESS)

(b) *fail:*SUCCESS(*em*_1_*,1,negative,em*_2_) ^ *stimulate(em*_2_*...)*

In the shared task context, the embedding predications of MODAL semantic type are most relevant to the speculation/negation detection task.

**Definition 9**. A modal predication, Pr_MODAL_, associates the predication it embeds, Pr*_e_*, with a **modality value **on a context-dependent **scale**. The scale (S) is determined by the semantic type of the modal predicate, P_MODAL_. The modality value (MV*_S_*) is a numerical value between 0 and 1 and corresponds to how strongly Pr*_e _*is associated with the scale S, 1 indicating strongest association and 0 negative association.

The scalar modality value is partially modeled after the modality value proposed by Nirenburg and Raskin [21]. In this view, a modality value of zero on the EPISTEMIC scale, for example, corresponds to "The speaker does not believe that *P*", while a value of 0.6 roughly indicates that "The speaker believes that possibly *P*". More often, modality values are represented discretely, when a single modality-related phenomenon is investigated (*certain*, *possible*, *probable *etc. on the *factuality *scale [16,17,22]). In our framework, we favor a contextual scale rather than a fixed one since it is more general and flexible.

#### ATTRIBUTIVE type

**Definition 10**. An **attributive **predicate links an embedded predication with one of its semantic arguments and specifies its semantic role.

Consider the fragment in Example (8a). The verbal predicate (*undergo*) takes a nominalized predicate (*degradation*) as its syntactic object. The other syntactic argument of the verbal predicate, *p105*, serves as the semantic argument of the embedded predicate (*degradation*) with the semantic role PATIENT. Example (8b) corresponds to the representation after the composition phase and Example (8c) shows the result of the mapping phase.

(8)   (a) *... p105undergoes**degradation **...*

(b) *p105:*PROTEIN*(t*_1_*) *^ *undergo:*PATIENT*(em*_1_*,e*_1_*,t*_1_*) *^ *degradation:*PROTEIN_ CATABOLISM*(e*_1_,_-_*)*

(c) *p105:*PROTEIN*(t*_1_*) *^ *degradation:*PROTEIN_CATABOLISM*(e*_1_*,t*_1_*)*

Verbs functioning in this way are plenty (e.g., *perform *corresponding to AGENT role, *experience *to EXPERIENCER role) [23]. Derivational forms of these verbs also function in the same way (e.g., *p105's undergoing of degradation*). With respect to the shared task, we found that the usefulness of the ATTRIBUTIVE type of embedding was largely limited to two verbal predicates, *involve *and *require*, and their nominalizations.

#### RELATIONAL type

**Definition 11**. A **relational **predicate semantically links two predications, providing a discourse/coherence function between them.

Discourse/coherence relations, discussed in various discourse models (e.g., Rhetorical Structure Theory [24], Penn Discourse TreeBank [25]), are typically indicated by syntactic classes such as subordinating and coordinating conjunctions (e.g., *although *and *and*, respectively), or discourse adverbials (e.g., *then*). However, they may also permeate to the subclausal level, often signalled by "discourse verbs" [26] (e.g., *cause*, *mediate*, *lead*, *correlate*), their nominal forms or other abstract nouns, such as *role*. These subclausal realizations appear frequently in biological research articles. We subcategorize the RELATIONAL type into CAUSAL, TEMPORAL, CORRELATIVE, COMPARATIVE, and SALIENCY types. We exemplify subclausal realizations of these categories in the shared task corpus below (See Figure 2 for the relevant logical forms for the sentence in Example (9a)):

(9)   (a) ***Stimulation ****of cells leads to a rapid **phosphorylation **of IκBα, which is presumed to be important for subsequent**degradation***. (CAUSAL, SALIENCY, and TEMPORAL, respectively)

(b) *This **increase **in p50 homodimers coincides with an **increase **in p105 mRNA, ... coincide:*CORRELATIVE(*em*_1_*,0.5,positive,em*_2_*,em*_3_) ^ *increase(em*_2_*...) *^ *increase(em*_3_*...)*

(c) ***Cotransfection ****with ... expression vectors produced a 5-fold increase compared with **cotransfection **with the ... expression vectors individually.*

*compare:*COMPARATIVE(*em*_1_*,1,positive,em*_2_*,em*_3_) ^ *cotransfection(em*_2_*...) *^ *cotransfection(em*_3_*...)*

Not all the subtypes of this class were relevant to the shared task: for example, comparative predications are not of interest. However, we found that CAUSAL, CORRELATIVE, and SALIENCY subtypes play a role, particularly in complex regulatory events.

#### VALENCE_SHIFTER type

**Definition 12. Valence shifting **describes the sentiment or polarity shift in a clause engendered by particular words, called **valence shifters **[27].

Three types of valence shifters are generally defined: NEGATOR (e.g., *not*), INTENSIFIER (e.g., *strongly*), and DIMINISHER (e.g., *barely*) [27-29].The type of embedding introduced by such words is crucial in semantic composition, as they behave similarly to MODAL predicates in changing the *scalar modality value *associated with the embedded predication. In Example (10a), the negative determiner *no *makes the binding event indicated by the verbal predicate *bound *non-factual. Example (10b) illustrates a diminishing effect, introduced by the adverb *slightly*.

(10)   (a) *... no NF-κB **bound **to the main NF-κB-binding site 2 of the IL-10 promoter ...*

(b) *FOXP3 was only slightly**reduced **after RUNX1 silencing*.

In the shared task setting, this type of embedding plays a role in speculation and negation detection.

### Dictionary of trigger expressions

Our methodology relies on a trigger dictionary, in which trigger expressions (predicates) are mapped to relevant atomic or embedding predication types. Previously, we relied on training data and simple statistical measures to identify good trigger expressions for biological event types and used a list of triggers that we manually compiled for speculation and negation detection (see [19,20] for details).

We currently take a more nuanced approach to trigger expressions to allow compositional analysis and characterize more subtle meaning distinctions. In contrast to our prior approach, we also allow multi-word triggers to some extent. Several entries from the trigger dictionary are summarized in Table 2. In the dictionary of trigger expressions, each predicate entry has six features:

**Table 2:** Embedding trigger dictionary entries

Predicate	POS	Semantic Type	Polarity	Category Strength	Negative-raising
*show*	VB	DEMONSTRATIVE	*positive*	1.0	false

*unknown*	JJ	EPISTEMIC	*negative*	0.7	false

*induce*	VB	CAUSAL	*positive*	1.0	false

*fail*	VB	SUCCESS	*negative*	1.0	false

*effect*	NN	CAUSAL	*neutral*	0.5	false

*weakly*	RB	DIMINISHER	*neutral*	1.0	false

*absence*	NN	NEGATOR	*negative*	1.0	false

Several entries from the embedding trigger dictionary.

***Lemma ***The lemma of the trigger expression.

***Part-of-speech ***The POS tag of the trigger.

***Semantic type ***One or more atomic/embedding predicate types.

***Polarity ***Whether the meaning contribution of the predicate is *positive*, *negative*, or *neutral*. For instance, with respect to the DYNAMIC:POTENTIAL category, the adjectival predicate *capable *has *positive *polarity, while the polarity of *unable *is *negative*.

***Category strength ***How strongly the trigger is associated with its semantic type. For example, the evidential predicate *show *is more strongly associated with the EVIDENTIAL:DEMONSTRATIVE category than the predicate *suggest*.

***Negative raising ***Whether the trigger allows transfer of negation to its complement. For example, *think*, *believe *allow negative raising. (*I don't think P *≡ *I think *¬*P*).

Polarity, category strength and negative raising features interact with semantic types to associate a context-dependent *scalar modality value *with predications, as indicated earlier. We denote the value of a feature *F *of a trigger P as *F*(P) (e.g., *Lemma*(P), *Sem*(P)).

The semantic types of atomic predicates are simply shared task event types determined from training data using maximum likelihood estimation, as before [19,20]. Using event types as semantic types of atomic predicates reflects our hypothesis that atomic predications are concerned with domain-specific events. Polarity values of atomic predicates are by default *neutral*, unless the trigger involves an affix which explicitly has positive or negative polarity (e.g., *nonexpression *(negative), *upregulation *(positive)). Category strength is simply set to 1, and negative raising is *false *by default.

On the other hand, we have been independently extending our manually compiled list of speculation/negation triggers to include other types of embedding predicates and to encode finer grained distinctions in terms of their categorization and trigger behaviors. This portion of the dictionary is composed of: (a) expressions compiled from relevant literature and linguistic classifications, (b) expressions automatically extracted from the shared task corpus as well as the GENIA event corpus [3], (c) limited extension based on lexical resources, such as WordNet [30] and UMLS Specialist Lexicon [31]. Some polarity values are derived from a polarity lexicon [32] and extended by using heuristics involving the predicate. For example, if the most likely event type associated with the predicate is NEGATIVE_REGULATION in the shared task corpus, we assume its polarity to be *negative*. Others are assigned manually. Similarly, some category strength values are based on our prior work [33], while others were manually assigned.

The trigger dictionary incorporates ambiguity; however, for the shared task, we limit ourselves to *one semantic type per predicate *to avoid the issue of disambiguation. For ambiguous triggers extracted from the training data, the semantic type with the maximum likelihood is used. This works well in practice, since the distribution of event types for a trigger word is generally skewed in favor of a single event type [20]. On the other hand, we manually determined the semantic type to use for triggers that we compiled independently of the training data. In this way, we use 466 atomic predicates and 908 embedding ones. All atomic predicates and 152 of the embedding predicates are drawn specifically from the shared task corpus.

### Composition phase

As mentioned above, the composition phase builds on simple entities, syntactic dependency relations and a trigger dictionary. Using these elements, we first construct a semantic embedding graph representing the content of the document, making *semantic dependencies *explicit. Entity semantics are provided in the shared task annotations. To obtain syntactic dependency relations, we segment each document into sentences, parse them using the re-ranking parser of Charniak and Johnson [34] adapted to the biomedical domain [35] and extract syntactic dependencies from the resulting parse trees using the Stanford dependency parser [36], which also provides token information, including lemma and positional information. We use the default Stanford dependency representation, *collapsed dependencies with propagation of conjunct dependencies*. We consult the trigger dictionary to identify predicate mentions in the document. After the semantic embedding graph for a document is constructed, we compose predications by traversing the graph in a bottom-up manner. We present a high level description of the composition phase below.

#### From syntactic dependencies to semantic embedding graph

We convert syntactic dependencies into a directed acyclic semantic embedding graph whose nodes correspond to *surface elements *of the document and whose labeled arcs correspond to semantic *embedding relations *between surface elements.

**Definition 13**. An **embedding relation ***E *holds between two surface elements *A *and *B *and has type *T*.

E:=T(A,B)

The surface element A is said to syntactically embed (or **s-embed**) B.

$$A{>}_{s}B$$

If the surface elements A and B are semantically bound, the semantic object associated with A embeds (and scopes over) that associated with B.

$$\left(A{>}_{s}B\right)\wedge \left[\left[A\right]\right]\neq \emptyset \wedge \left[\left[B\right]\right]\neq \emptyset \Rightarrow \left[\left[A\right]\right]>\left[\left[B\right]\right]$$

An embedding relation is clearly similar to a syntactic dependency. However, in contrast to a syntactic dependency, direction of an embedding relation reflects the semantic dependency between its elements, rather than a syntactic one, and a semantic dependency can cross sentence boundaries. We distinguish embedding relations from syntactic dependencies by capitalizing their types (labels).

A set of intra-sentential transformation rules, illustrated in Table 3, take syntactic dependencies, entity and predicate mentions of a sentence, and identify surface elements and intra-sentential embedding relations. Consider the first row in Table 3, where the focus is on the noun phrase *CD40 ligand interactions*. An entity and a predicate mention (*CD40 ligand *and *interactions*, respectively) are associated with this noun phrase. The corresponding transformation rule (NP-Internal Transformation) aims to identify semantic dependencies within a noun phrase. As illustrated in Table 3, two syntactic dependencies exist between the tokens of the noun phrase, both *nn *(nominal compound modifier) dependencies between the head and a modifier. The modifiers correspond to the entity mention. This transformation, then, collapses the modifiers, allowing us to treat them as a single, semantically bound surface element. It also collapses two syntactic dependencies into one embedding relation between the head and the newly formed surface element.

**Table 3:** Application of intra-sentential transformation rules

Fragment	Syntactic Dependencies	Embedding Relations
*... CD40 ligand interactions play a key role ...*	*nn(interactions,ligand) nn(interactions,CD40)*	*NN(interactions, CD40 ligand)*

*... specifically binds and phosphorylates IκBα*	*conj*_*and(*binds,phosphorylates*)*	*CC(and, binds)* *CC(and, phosphorylates)*

*... possible involvement of HCMV ...*	*amod(involvement,possible) prep*_*of(involvement,HCMV)*	*AMOD(possible,involvement) PREP_OF(involvement, HCMV)*

*... Tat and Sp1 proteins ...*	*nn(proteins,Sp1)* *conj_and(Tat,proteins)*	*NN(proteins, and)* *CC(and,Tat)* *CC(and, Sp1)*

Application of several intra-sentential transformation rules to the sentence fragments in the first column. The syntactic dependencies in the second column are the input to these rules and the embedding relations in the third column are the output.

In addition to *collapsing *several syntactic dependencies into one embedding relation (row 1), a transformation rule may result in *splitting *one into several embedding relations (row 2) (Coordination Transformation), or in *switching *the direction of the dependency (row 3) (Dependency Direction Inversion). In addition to capturing semantic dependency behavior explicitly and incorporating semantic information (entity and predicate mentions) into the embedding structure, these transformations also allow us to correct syntactic dependencies that are systemically misidentified, such as those that involve modifier coordination (row 4) (Corrective Transformation). Also note that a transformation is not necessary when the syntactic dependency under consideration is isomorphic to an embedding relation, that is, it reflects the direction of the semantic dependency accurately (*prep*_*of *dependency in row 3). We currently use 13 such transformation rules, hand-crafted by analyzing the relevant syntactic constructions and the corresponding syntactic dependency configurations.

Once these intra-sentential transformations are complete, we finalize the document embedding graph by considering two types of special embedding relations:

***PREV ***A semantic dependency that holds between the topmost nodes associated with adjacent sentences as to reflect the sequence of sentences.

***COREF ***A coreference relation that holds between an anaphoric element and its antecedent. The antecedent may be in the same sentence as the anaphor or in a prior sentence.

**Figure 4:**
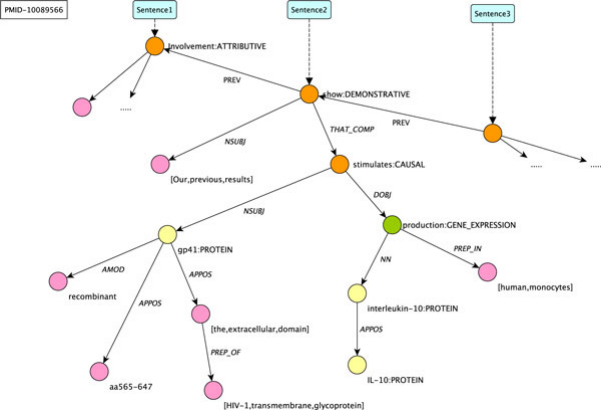
**An example embedding graph**. A portion of the embedding graph associated with a Medline abstract (10089566). The sentence under consideration is *Our previous results show that recombinant gp41 (aa565-647), the extracellular domain of HIV-1 transmembrane glycoprotein, stimulates interleukin-10 (IL-10) production in human monocytes*. Yellow circles represent surface elements bound to PROTEIN entities, green circles those bound to atomic predicates, and the orange circles to embedding predicates.

These special relations allow us to address event extraction beyond the sentence level. We will turn to coreference resolution later at the end of this section. A portion of an example document embedding graph is given in Figure 4.

#### Composing predications

After constructing the document embedding graph, we traverse it in a bottom-up manner and compose predications. At this stage, it is important to remember that we refer to the revised definition of predication here, represented as follows, where POL is the *polarity value *and MV*_S _*is the *scalar modality value*.

$$Pr=:\left[P,MV_{S},POL,ARG_{1..n}\right]$$

The *polarity value *can be positive, negative or neutral. For simplicity, we limit here *scalar modality values *to the [0, 1] range and compute it for a predication that is in the scope of a MODAL and VALENCE_SHIFTER predicate. Atomic predications initially take the polarity value assigned to their trigger in the dictionary and a modality value of 1.0.

**Definition 14**. An **argument identification rule ***R:Q*→A is a typing function. *Q *is a 4-tuple 〈*T, POS, IN, EX*〉, where

• *T *is an embedding relation type

• *POS *is a part-of-speech category

• *IN *and *EX *are sets denoting inclusion and exclusion constraints, respectively

and A is the set of logical argument types (A = {Object, Subject, Adjunct}). A predicate *P ***satisfies **a constraint *C *if its lemma or semantic type is included in *C*.

Lemma(P)∈C∨Sem(P)∈C⇒satisfies(P,C)

Let *V *be a surface element corresponding to a non-leaf node in the embedding graph and *E *an embedding relation, such that *E = T(V,V_e_)*. An argument identification rule *R *applies to the pair *(V,E) *and assigns the surface element *V_e _*as the logical argument of type *f *or the *V*, if

$$\begin{array}{c} Part\text{\_}of\text{\_}speech\left(V\right)=POS\wedge \left(satisfies\left(V,IN\right)\vee \left(\neg satisfies\left(V,IN\right)\wedge \neg satisfies\left(V,EX\right)\right)\right)\Rightarrow \\ \text{A(}V\text{) = }V_{e}\wedge applies\text{\_}to\left(R,V,E\right) \end{array}$$

Some argument identification rules are exemplified in Table 4. We currently use about 80 such rules, adapted and extended from our previous shared task system [19,20]. After all the children nodes of a non-leaf node are recursively processed for logical arguments, a predication can be composed. Composition involves three operations: *polarity composition*, *modality value composition*, and *argument propagation*.

**Table 4:** Argument identification rules

Embedding Relation Type	POS	Inclusions	Exclusions	Argument Type
*PREP*_*ON*	NN	*influence,impact,effect*	-	Object

*AGENT*	VB	-	-	Subject

*NSUBJPASS*	VB	-	-	Object

*WHETHER*_*COMP*	VB	INTERROGATIVE	-	Object

*PREP*_*IN*	NN	-	*effect,role,influence,importance*	Adjunct

Several argument identification rules. For a rule R:*Q*→A, where *Q *= 〈*T,POS,IN,EX*〉, column 1:*T*, column 2:*POS*, column 3: *IN*, column 4:*EX*, and column 5:A. Note that inclusion and exclusion constraints may apply to predicate categories, as well as to specific lemmas.

##### Polarity composition

*Polarity composition *is relevant in the context of embedding predications. The *polarity value *of such a predication depends on:

• The *polarity value *of its trigger (from the dictionary)

• The *embedded polarity value *associated with the embedded predication

Table 5 illustrates some of the polarity composition operations. An example is presented below, where the composite polarity value of *leads to the prevention *is determined to be negative (11d), from the information given in (11b).

**Table 5:** Polarity value composition

Predicate polarity	Embedded polarity value	Composite polarity
neutral	positive	positive

neutral	negative	negative

negative	*	negative

positive	negative	negative

positive	*	positive

The composition of polarity value of an embedding predication from polarity value of the predicate and embedded polarity value.

(11)   (a) *... Bcl-2 overexpression leads to the prevention of chemotherapy (paclitaxel)-induced expression...*

(b) *prep*_*to(leads,prevention)*

Polarity(lead) = positive

Polarity(prevention) = negative

(c) *lead:*CAUSAL(*em*_1_*,1,positive,... em*_2_*...*) ^ *prevention:*CAUSAL(*em*_2_*,1,negative,... em*_3_) ...

(d) *lead*_*prevention:*CAUSAL(*em*_1_*,1,negative, ... em*_3_)

##### Modality value composition

*Modality value composition *is only relevant for MODAL and VALENCE_SHIFTER type predicates, because only these predicates have scale-shifting properties. When a predicate of one of these types is encountered during graph traversal, we percolate its modality effect down to update the *scalar modality values *of the predications in its scope. This procedure, illustrated in Example (12) below, is affected by several factors:

• The *semantic type *of the current predicate (*Sem*(P))

• Its *category strength *as specified in the dictionary (*Strength*(P))

• The *embedded scalar modality value *of the embedded predication (MV*_S_*(Pr*_e_*))

Let us consider the underlined fragments in Example (12a) to characterize modality value composition. The embedding relations between the underlined fragments are given in Example (12b) and syntactic embeddings in (12c).

(12)   (a) *Thus, ... IL-10 upregulation in monocytes may not involve NF-κB, MAPK, or PI 3-kinase activation, ...*

(b) *ADVMOD(Thus,may)*

AUX(may,not)

NEG(not,involve)

NSUBJ(involve,upregulation)

(c) *Thus >_s _may >_s _not >_s _involve >_s _upregulation*

Valence shifting and modality are encoded with *not *and *may *nodes in the graph, respectively. They affect the scalar modality value directly: *not *changes the modality value of the predication bound to its child, *involve*, from 1 (default modality value for the predicate *involve*) to 0, since it is a negative valence shifter (Definition 15).

**Definition 15**. A predicate P, associated with embedding predication Pr, **inverses **the modality value of the predication it embeds, Pr*_e_*, with respect to [0, 1] range, if it is a negative valence shifter.

$$Sem(P)=\mathrm{NEGATOR}\wedge (Pr>Pr_{e})\Rightarrow MV_{S}{\left(Pr_{e}\right)}^{\prime }=1-MV_{S}(Pr_{e})$$

The *may *node, parent of the *not *node, shifts the modality value of Pr*_e _*from 0 to 0.3 (see Definition 16). Note that *may *is a predicate of SPECULATIVE type and has a category strength of 0.7. The increase illustrates the fact that while a modal predicate like *may *normally lowers the modality value of an embedded predication in a positive context, its effect is to increase this value when the embedded predication is initially in a negative context (*not involve*).

**Definition 16**. A MODAL predicate P, associated with embedding predication Pr, lowers the modality value of the predication it embeds, Pr*_e_*, proportional to its category strength, if MV*_S_*(Pr*_e_*) is initially closer to 1 on scale.

$$Sem(P)=\text{MODAL}\wedge (Pr>Pr_{e})\wedge (MV_{S}(Pr_{e})\geq 0.5)\Rightarrow MV_{S}{\left(Pr_{e}\right)}^{\prime }=Strength(P)*MV_{S}(Pr_{e})$$

Otherwise, P increases MV*_S_*(Pr*_e_*) proportional to its category strength.

$$\begin{array}{l} Sem(P)=\text{MODAL}\wedge (Pr>Pr_{e})\wedge (MV_{S}(Pr_{e})<0.5)\Rightarrow MV_{S}{\left(Pr_{e}\right)}^{\prime }=\\ MV_{S}(Pr_{e})+(1-Strength(P))*(1-MV_{S}(Pr_{e}) \end{array}$$

Polarity and modality value composition is inspired by studies exploring compositional approaches to sentiment analysis or textual entailment tasks [27,37,38].

##### Argument propagation

*Argument propagation *is concerned with determining whether a descendant of the current node can serve as its argument, when the intermediate nodes between them are *semantically free*.

**Definition 17**. Let *A *and *C *be semantically bound surface elements (⟦*A*⟧ ≠ ∅ ^ ⟦*C*⟧ ≠ ∅), *C *an ancestor of *A *in the embedding graph, and  the set of nodes that form the path from *C *to *A *(ℬ≠∅). ⟦*A*⟧ can be an argument to ⟦*C*⟧, if all nodes on the path are semantically free and there is an embedding relation *E *such that *E = T(C,B_i_)*, where $B_{i}\in ℬ$, and an argument identification rule R applies to the *(C,E) *pair:

$$E=T\left(C,B_{i}\right)\wedge B_{i}\in ℬ\wedge \forall B:\left(B\in ℬ\wedge \left[\left[B\right]\right]=\emptyset \right)\wedge \exists R:applies\text{\_}to\left(R,C,E\right)$$

Consider the sentence in Example (13a). The entities associated with the fragment are underlined, the embedding relations are given in (13b), and the result of the composition in (13c).

(13)   (a) *... no NF-κB **bound**_C _to the main NF-κB-binding site_B _2 of the IL-10_A _promoter after addition of gp41*.

(b) *PREP*_*TO(bound,site)*

*PREP*_*OF(site,promoter)*

NN(promoter,IL-10)

*PREP*_*AFTER(bound,addition)*

*PREP*_*OF(addition,gp41)*

(c) *bind:*BINDING*(e*_1_*,t*_1_*) *^ *IL-10:*PROTEIN*(t*_1_)

When traversing the embedding graph, checking the daughter nodes of the node *bound *(corresponding to C in Definition 17) for arguments invokes an argument identification rule, which stipulates that *bind *can link to an argument of Object type via an embedding relation of *PREP*_*TO *type, which in this case is *site *(*B*), a nonentity. At this point, argument propagation makes the nodes in scope of the daughter node accessible, which results in finding the node *IL-10 *(*A*), corresponding to a PROTEIN term. Thus, *IL-10 *is allowed as an Object argument of *bound*. On the other hand, another semantically bound node, *gp41*, cannot be an argument of *bound*, since the type of the relevant embedding relation is *PREP*_*AFTER*, which does not license an argument identification rule.

Besides these compositional operations, this phase also deals with coordination of entities and triggers. This phase results in a set of predications, forming a directed acyclic graph of fully composed predications. For the sentence depicted in Figure 4, duplicated in (14a), the relevant resulting embedding and atomic predications are given in (14b). Note that the first argument corresponds to Object, the second to Subject, and the rest to Adjunct arguments.

(14)   (a) *Our previous results show that recombinant gp41 (aa565-647), the extracellular domain of HIV-1 trans-membrane glycoprotein, stimulates interleukin-10 (IL-10) **production **in human monocytes*.

(b) *show:*DEMONSTRATIVE(*em*_1_*,1,positive,em*_2_,t_3_) ^ *our-previous-result(t*_3_*)*

*stimulate:*CAUSAL(*em*_2_*,1,positive,e*_2_*, t*_1_) ^ *gp41:*PROTEIN*(t*_1_*)*

*production:*GENE_EXPRESSION(*e*_2_*,t*_2_,_-_,t_4_) ^ *interleukin-10:*PROTEIN*(t*_2_*) *^ *human-monocyte(t*_4_*)*

### Mapping predications to events

The *mapping *phase imposes shared task constraints on the partial interpretation obtained in the composition phase. We achieve this in three steps.

The first step of the mapping phase is to convert embedding predication types to event (or event modification) types. This step is guided by constraints on embedding predication type, polarity and modality values, as presented in Table 6. In this way, *em*_1 _in Example (14a) is pruned, since it has positive polarity. As the constraint in the last row of Table 6 illustrates, embedding predications of DEMONSTRATIVE type are relevant to the shared task only when they have negative polarity, that is, when they indicate *lack of proof *and, thus, speculation.

**Table 6:** Mapping from embedding predications to events

Track	PredicationType	Polarity	Modality Value	Correspond. Event (Mod.) Type
GENIA,ID	CAUSAL	neutral	-	REGULATION

GENIA,ID,EPI	SUCCESS	negative	-	NEGATION

EPI	CAUSAL	positive	-	CATALYSIS

GENIA,ID,EPI	SPECULATIVE	-	*>*0.0	SPECULATION

GENIA,ID,EPI	DEMONSTRATIVE	negative	-	SPECULATION

Constraints used in mapping from embedding predication types to event and event modification types.

Next, we convert logical arguments to semantic roles. A small number of mappings, illustrated in Table 7, are defined for this purpose. These are similar to *argument identification rules*, in that the mapping can be constrained to certain event types or event types can be excluded from it. For example, the first two mappings (row 1-2) allow the Object and Subject arguments of *em*_2 _in Example (14b) to be converted to Theme and Cause semantic roles, respectively.

**Table 7:** Mapping logical arguments to semantic roles

Logical Argument	Constrained To	Exclusions	Semantic Role
Object	-	PROCESS	Theme

Subject	-	BINDING	Cause

Subject	BINDING	-	Theme

Object	PROCESS	-	Participant

Object	SPECULATION, NEGATION	-	Scope

Logical argument to semantic role mappings.

Finally, we prune event participants that do not conform to the event definition or are semantically free as well as the predications whose types could not be mapped to a shared task event type. Thus, a Cause participant for a GENE_EXPRESSION event is pruned, since only Theme participants are annotated as relevant for the shared task; likewise, a predication with DEONTIC semantic type is pruned, because such predications are not considered for the shared task. Furthermore, the adjunct argument of the GENE_EXPRESSION event (*t*_4_) is pruned since (a) it is semantically free, and (b) we are not dealing with non-core arguments at the moment. The Infectious Diseases track (ID) event type PROCESS is exceptional, because it may take no participants at all, and we deal with this idiosyncrasy at this step, as well. This concludes the progressive transformation of the graph to event and event modification annotations. The annotations corresponding to the predications in Example (14) are given below. Note that triggers are not shown as separate term annotations for simplicity.

(15)   (a) E1 Positive_regulation:*stimulates *Theme:E2 Cause:T1

(b) E2 Gene_expression:*production *Theme:T2

### Coreference resolution

The inability to resolve coreference has emerged as a factor that hindered event extraction in the BioNLP'09 Shared Task on Event Extraction [39]. Coreference resolution is essentially a recall-increasing measure: in the following fragment, recognizing that *Eotaxin *is the antecedent of the pronominal anaphor *Its*, would allow our system to identify this term as the Theme participant of the GENE_EXPRESSION event triggered by the nominalization *expression*, which would remain unidentified otherwise.

(16)   (a) *Eotaxin is an eosinophil specific beta-chemokine assumed to be involved in eosinophilic inflammatory diseases such as atopic dermatitis, allergic rhinitis, asthma and parasitic infections. Its**expression **is stimulus- and cell-specific*.

(b) *expression:*GENE_EXPRESSION*(e*_1_*,t*_1_*) *^ *eotaxin:*PROTEIN*(t*_1_*)*

The Protein Coreference Task [10] was proposed as a supporting task in BioNLP'11-ST. The performance of participating systems in this supporting task were not particularly encouraging with regard to their ability to support event extraction, with the best system achieving an F_1_-score of 34.05 [40]. Post-shared task, we extended our embedding framework with coreference resolution and examined the effect of different classes of anaphora on event extraction. In the description of the Protein Coreference Task [10], four main classes of coreference are identified:

**RELAT **Coreference indicated by relative pronouns and adjectives (e.g., *that, which, whose*)

**PRON (*pronominal anaphora*) **Coreference indicated by personal and possessive pronouns (e.g., *it*, *its*, *they*, *their*)

**DNP (*sortal anaphora*) **Coreference indicated by definite and demonstrative noun phrases (NPs that begin with *the*, *these*, *this*, etc.)

**APPOS **Coreference in appositive constructions

Our embedding framework performs coreference resolution as a subtask of the composition phase. It accommodates RELAT and APPOS classes naturally, since they are intra-sentential and they can largely be identified based on embedding relations alone. For the more complex anaphoric classes (PRON and DNP), we extended our framework. Our extension is partially inspired by the deterministic coreference resolution system described in Haghighi and Klein [41]. To summarize, for each anaphoric mention identified in the text, their system selects an antecedent among the prior mentions by utilizing syntactic constraints and assessing the semantic compatibility between mentions. Of the remaining possible antecedents, the one with the shortest path from the anaphoric mention in the parse tree is selected as the best antecedent. The syntactic constraints used by their system include number, person, and entity type agreement as well as recognition of appositive constructions. On the other hand, their semantic compatibility filter aims to pair hypernyms, such as *AOL *and *company*. They extract such pairs from their corpus using bootstrapping. We provide more details about our treatment of the four coreference classes below.

#### RELAT and APPOS type

The RELAT type is the most frequent type of coreference annotated for the Protein Coreference Task (56% of all training instances), while the APPOS type is rarely annotated. To determine the antecedent *ANT *of a relative pronoun *RP*, we use the following transformation rule, where *rel *denotes a *relative *dependency, and *rcmod *a *relative clause modifier *dependency. This rule simply states the antecedent of a relative pronominal anaphora is the noun phrase head it modifies.

rel(X,RP)∧rcmod(ANT,X)⇒COREF(RP,ANT)

On the other hand, coreference in appositive constructions is handled with the following rule, where *APPOS *∈ {*appos*, *abbrev*, *prep*_*including*, *prep*_*such*_*as*}.

APPOS(ANT,ANA)∨APPOS(ANA,ANT)⇒COREF(ANA,ANT)

#### PRON and DNP type

PRON type of coreference is the second most frequent type of coreference annotated for the Protein Coreference Task (35% of all training instances), while the DNP type corresponds to 9% of the training instances. With respect to the PRON type, we only consider personal and possessive pronouns of the third person (*it/its*, *they/their*) as anaphora, since others do not seem relevant to the event extraction task (e.g., *Our results*). For sortal anaphora, the DNP type, we require that the anaphoric noun phrases are not associated with entities, allowing expressions such as *these factors *as anaphora while ruling out those like *the TRADD protein*.

Coreference resolution begins by identifying the set of candidate antecedents. We define the candidate antecedent set for a given anaphor as the set of embedding graph nodes which appear in the discourse prior to the anaphor and which are either semantically bound or involve hypernyms or conjunctions. The prior discourse includes the sentence that the anaphora occurs in as well as those preceding it in the paragraph.

The candidate antecedents are then evaluated for their syntactic and semantic compatibility. PRON requires person and number agreement, while DNP requires number agreement and one of the following constraints:

***The head word constraint ***The head of the anaphoric NP and the antecedent NP are the same. This constraint allows "*CD4 gene*" as an antecedent for the anaphor "*the gene*".

***The singular hypernymy constraint ***The head of the anaphoric NP is a hypernym of the antecedent, which involves an entity. This constraint accepts any Protein term as an antecedent for the anaphoric NP "*this protein*".

***The plural hypernymy constraint *(*set-instance anaphora*) **The head of the anaphoric NP is a plural hypernym of the antecedent, which corresponds to a conjunction of entities. This constraint accepts "*CD1, CD2, and CD3*" as antecedent for "*these factors*".

***The meronymy constraint ***The head of the anaphoric NP is a meronym and the antecedent corresponds to a conjunction of entities. This constraint allows "*IBR/F*" as antecedent for the anaphoric NP "*the dimer*".

***The event constraint ***The head of the anaphoric NP is associated with a trigger, P_1_, and the antecedent with another trigger, P_2_, where P_1 _and P_2 _are lexicalizations of the same event. This constraint aims to capture the coreference between, for instance, the anaphor *the phosphorylation *and the antecedent *phosphorylated*.

We induced the hypernym list from the training corpus automatically by considering the heads of the NPs with entities in modifier position. Such words include *gene*, *protein*, *factor*, and *cytokine*. Similarly, we induced the meronym list from the training data of the Static Relations supporting task [11]. These words essentially correspond to triggers for SUBUNIT-COMPLEX relations in that task, and include words such as *complex*, *dimer*, and *subunit*.

Several structural constraints over the embedding graph block some of the possible antecedents for both coreference types:

• The antecedent directly embeds or is directly embedded by the anaphor.

• The antecedent is the subject and the anaphor is the object of the same relation. In addition, the anaphor is not reflexive (e.g., *itself*).

• The anaphor is in an adjunct position and the antecedent is in subject position of the same relation.

The candidate that is closest to the anaphor in the embedding graph is selected as the antecedent and a *COREF *embedding relation is created between the anaphor and the antecedent. For plural anaphora, multiple entities or triggers may be considered as antecedents, and thus multiple *COREF *relations may be created.

The integration of coreference information into the event extraction pipeline is trivial for all coreference types. In the composition phase, when an anaphoric expression appears in the argument position of a predication, it is naturally substituted by its antecedent(s) through argument propagation.

## Results and discussion

With the two-phase methodology presented above, we participated in three tracks: GENIA (Tasks 1 and 3), ID, and EPI. The official evaluation results we obtained for the GENIA track are presented in Table 8 and the results for the EPI and ID tracks in Table 9. With the official evaluation criteria, we were ranked 5th in the GENIA track (5/15), 7th in the EPI track (7/7) and 4th in the ID track (4/7). There were only two submissions for the GENIA speculation/negation task (Task 3) and our results in this task were comparable to those of the other participating group [42]; our system performed slightly better with speculation, and theirs with negation. We note that their system was ranked higher than ours in Task 1 (3rd), which suggests that our system performance on speculation/negation task *alone *is probably a bit better than theirs. For full comparison with the other participating systems, we refer the reader to the shared task overview papers [10,11].

**Table 8:** Official GENIA track results

Event Class	Recall	Precision	F_1_-score	Rank
Localization	39.27	90.36	54.74	7

Binding	29.33	49.66	36.88	7

Gene_expression	65.87	86.84	74.91	5

Transcription	32.18	58.95	41.64	9

Protein_catabolism	66.67	71.43	68.97	2

Phosphorylation	75.14	94.56	83.73	4

EVT-TOTAL	52.67	78.04	62.90	6

Regulation	33.77	42.48	37.63	3

Positive_regulation	35.97	47.66	41.00	7

Negative_regulation	36.43	43.88	39.81	5

REG-TOTAL	35.72	45.85	40.16	5

Negation	18.77	44.26	26.36	2

Speculation	21.10	38.46	27.25	1

MOD-TOTAL	19.97	40.89	26.83	2

ALL-TOTAL	43.55	59.58	50.32	5

Official GENIA track results, with the *approximate span matching/approximate recursive matching *evaluation criteria.

**Table 9:** Official EPI and ID track results

Track-Eval. Type	Recall	Precision	F_1_-score	Rank
EPI-FULL	20.83	42.14	27.88	7

EPI-CORE	40.28	76.71	52.83	6

				

ID-FULL	49.00	40.27	44.21	4

ID-CORE	50.91	43.37	46.84	4

				

ID-FULL-T	45.26	53.18	48.90	4

ID-CORE-T	46.75	56.94	51.34	4

Official evaluation results for EPI and ID tracks. The primary evaluation criteria underlined. ID-FULL-T and ID-CORE-T refer to the post-shared task scenario where ID triggers are drawn only from ID training data.

### Development set vs. test set

A particularly encouraging outcome for our system is that our results on the GENIA development set versus on the test set were very close (an F_1_-score of 51.03 vs. 50.32), indicating that our general approach avoided overfitting, while capturing the linguistic generalizations, as we intended. We observe similar trends with the other tracks, as well. In the EPI track, development/test F_1_-score results were 29.1 vs. 27.88; while, in the ID track, interestingly, our test set performance was better (39.64 vs. 44.21). We also obtained the highest recall in the ID track (49), despite the fact that our system typically favors precision. We attribute this somewhat idiosyncratic performance in the ID track partly to the fact that we did not use a track-specific trigger dictionary for the official submission. All but one of the ID track event types are the same as those of the GENIA track, which led to identification of some ID events with triggers consistently annotated only in the GENIA corpus and to low precision particularly in complex regulatory events. A post-shared task re-evaluation confirms this: the F_1_-score for the ID track increases from 44.21 to 48.9 when only triggers extracted from the ID track corpus are used; recall decreases from 49 to 45.26, while the precision increases from 40.27 to 53.18. It is unclear to us why a reliable trigger in one corpus is not reliably annotated in another, even though the same event types are considered in both corpora. One possibility is that different annotators may have a different conceptualization of the same event types. Consider the following sentences: Example (17a) is from the GENIA corpus and Example (17b) from the ID corpus. Even though the verbal predicate *lead *appears in similar contexts in both sentences, it is annotated as an event trigger only in Example (17a).

(17)   (a) *Costimulation of T cells through both the Ag receptor and CD28 leads to high level IL-2 **production **...*

*lead:*POSITIVE_REGULATION(*em*_1_*,em*_2_)

*high*_*level:*POSITIVE_REGULATION(*em*_2_*,e*_3_)

*production:*GENE_EXPRESSION(*e*_3_*,t*_1_) ^ *IL-2:*PROTEIN*(t*_1_*)*

(b) *... the two-component regulatory system PhoR-PhoB leads to increased hilE P2 **expression **...*

*increased:*POSITIVE_REGULATION(*em*_1_*,e*_2_*,t*_1_) ^ *PhoR-PhoB:*PROTEIN*(t*_1_*)*

*expression:*GENE_EXPRESSION(*e*_2_*,t*_2_) ^ *hilE:*PROTEIN*(t*_2_*)*

We refer to the results concerning the post-shared task re-evaluation as ID-T in Tables 9 and 12.

### Full-text articles vs. abstracts

One of the interesting aspects of the shared task was its inclusion of full-text articles in training and evaluation. Cohen et al. [43] show that structure and content of biomedical abstracts and article bodies differ markedly and suggest that some of these differences may pose problems in processing full-text articles. Since one of our goals was to determine the generality of our system across text types, we did not perform any full text-specific optimization. Our results on article bodies are notable: our system had stable performance across text types (in fact, we had a very slight F_1_-score improvement on full-text articles: 50.28 to 50.4). This contrasts with the drop of a few points that seems to occur with other well-performing systems. Taking only full-text articles into consideration, we would be ranked 4th in the GENIA track. Furthermore, a preliminary error analysis with full-text articles indicates that parsing-related errors are more prevalent in the full-text article set than in the abstract set, consistent with Cohen et al.'s [43] findings. At the same time, our results confirm that we were able to abstract away from such errors by a careful, selective use of syntactic dependencies and correcting them with heuristic transformation rules, when necessary.

### Cause participants of regulatory events

The regulatory events in the GENIA track may take Cause arguments as core participants. They are annotated much less frequently than the other core argument, Theme, and therefore, it may be more challenging for machine-learning based methods to extract Cause arguments than to extract Theme arguments. Since our methodology is less reliant on the training data with respect to argument identification, we find it informative to compare our results in identifying Cause participants to the results of other systems. The comparison reveals that our system performs the best in identifying the Cause participants (F_1_-score of 43.71), confirming our intuition that linguistically-grounded methods may perform better in the absence of large amounts of annotated data.

### Non-core event participants

Our core module can extract adjunct arguments, using ABNER [44] as its source for additional biological named entities. We experimented with mapping these arguments to non-core event participants (Site, toLoc, etc.); however, we did not include them in our official submission, because they seemed to require more work with respect to mapping to shared task specifications. Due to this shortcoming, the performance of our system suffered significantly in the EPI track, in which the primary evaluation criterion involves non-core event participants as well as the core participants.

### Speculation and negation

Speculation and negation are most closely associated with our *embedding *focus. Therefore, we examined our results on the GENIA development set with respect to speculation and negation detection (Task 3) more closely. Consistent with our previous shared task results, we determined that the majority of errors were due to misidentified or missed base events (70% of the precision errors and 83% of the recall errors). An even bigger percentage of speculation/negation-related errors in the EPI and ID tracks were due to the same problem, as the overall accuracy in those tracks is lower. When we use the gold standard GENIA event annotations as input to the system and, thus, eliminate Task 1-related errors and evaluate speculation/negation detection *alone*, we obtain the results shown in Table 10. These results constitute a more accurate characterization of the system in speculation/negation detection than the official results, which do not account for Task 1-related errors.

**Table 10:** GENIA Task 3 results based on gold event annotations

Event Modification Type	Recall	Precision	**F**_1_**-score**
NEGATION	49.31 (18.77)	87.70 (44.26)	63.13 (26.36)

SPECULATION	65.70 (21.10)	73.27 (38.46)	69.28 (27.25)

MOD-TOTAL	57.95 (19.97)	78.47 (40.89)	66.67 (26.83)

Task 3 results when gold standard event annotations are provided to the system. Official results are duplicated in parentheses for reference.

Task 3-specific precision errors included cases in which speculation or negation was debatable, as the examples below show. In Example (18a), our system detected a SPECULATION instance, due to the verbal predicate *suggesting*, which scopes over the event indicated by *role*. In Example (18b), our system detected a NEGATION instance, due to the verbal predicate *lack*, which scopes over the events indicated by *expression*. Neither were annotated as such in the shared task corpus. Annotating negation and speculation is clearly nontrivial, as there seems to be some subjectivity involved, and such errors seem acceptable to a certain extent.

(18) (a) *... suggesting a **role **of these 3' elements in beta-globin gene expression*.

(b) *... DT40 B cell lines that lack**expression **of either PKD1 or PKD3 ...*

Another class of precision errors was due to argument propagation. The current algorithm appears to be too permissive in some cases and a more refined approach to argument propagation may be necessary. In the following example, while *suggest*, an epistemic predicate, does not s-embed *induction *(as shown in (19b)), the intermediate nodes simply propagate the predication associated with the *induction *node up the graph, leading us to conclude that the predication triggered by *induction *is speculated, leading to a precision error.

(19)   (a) *... these findings suggest that PWM is able to initiate an intracytoplasmic signaling cascade and EGR-1 **induction **...*

(b) *suggest >_s _able >_s _initiate >_s _induction*

Simply restricting argument propagation to one level increases the precision and F_1_-score slightly (from 66.67 to 66.93). Disallowing it altogether (that is, using the immediate daughters as arguments only), however, increases precision while lowering recall and F_1_-score significantly (from 66.67 to 61.31). This result indicates that the types of the embedding relations along the path from the trigger node to the target node play a larger role in determining whether the target node can act as an argument than the length of the path.

Some of the recall errors were due to shortcomings in the argument identification rules, as it is currently implemented. One recall problem involved the embedding status of and rules concerning copular constructions, which we had not yet addressed. Therefore, we miss the relatively straightforward SPECULATION instance in the following example.

(20)   *... the A3G promoter appears constitutively **active***.

Similarly, the lack of a trigger expression in our dictionary causes recall errors. One example below (21a) shows an instance where this occurs, in addition to lack of an appropriate argument identification rule, while the recall error in (21b) is solely due to the lack of the trigger expression:

(21)   (a) *mRNA was quantified by real-time PCR for FOXP3 and GATA3 **expression***.

(b) *To further characterize **altered **expression of TCRζ, p56(lck) ...*

Our system also missed an interesting, domain-specific type of negation, in which the minus sign acts similar to a negative determiner (e.g., *no*) and indicates negation of the event that the entity participates in.

(22)   *... CD14- surface Ag **expression **...*

### Coreference resolution

In the supporting Protein Coreference Task, we were ranked third (out of 6 participants) and achieved an F_1_-score of 29.65 by simply focusing on coreference of RELAT type. However, we find it more important to evaluate coreference resolution not in isolation but within the context of event extraction, in the spirit of Yoshikawa et al. [45], who improved the results of an event extraction system using coreference information. We measured the effect of each type of coreference resolution (RELAT, APPOS, PRON and DNP) on event extraction over the GENIA development set. The results, presented in Table 11, show that improvement in event extraction performance due to our current coreference resolution algorithm is modest. We observe that there is a consistent recall increase, while the precision suffers slightly in all cases. Resolving all four classes of coreference simultaneously seems to have a synergistic effect on the performance. On the test sets of the three tracks we participated in, we see minor improvements due to coreference resolution in GENIA and EPI tracks, but not in the ID track, as shown in Table 12.

**Table 11:** Coreference resolution on GENIA development set

System	Recall	Precision	**F**_1_**-score**
Base	46.32	56.81	51.03

Base + RELAT	46.57	56.52	51.06

Base + APPOS	47.07	56.40	51.32

Base + PRON	46.76	56.28	51.08

Base + DNP	46.85	56.26	51.13

Base + ALL	47.98	55.77	51.62

Effect of different types of coreference resolution on event extraction performance on GENIA development set with the *approximate span matching/approximate recursive matching *evaluation criteria.

**Table 12:** Coreference resolution on test sets

System	Recall	Precision	**F**_1_**-score**
GENIA	43.55	59.58	50.32
GENIA + COREF	44.45	58.92	50.67
- Abstracts	44.31	59.82	50.91
- Full-text	44.78	56.82	50.09

EPI	20.83	42.14	27.88
EPI + COREF	21.48	40.63	28.10

ID	49.00	40.27	44.21
ID + COREF	49.97	38.81	43.69
ID-T	45.26	53.18	48.90
ID-T + COREF	46.37	50.95	48.55

Event extraction performances after coreference resolution with the primary evaluation criteria.

It is interesting to note that while the APPOS type coreference was rarely annotated in Protein Coreference Task corpus, resolving it had the biggest effect on event extraction. This is in contrast to the RELAT type, which had the highest percentage of instances in the corpus but had little effect on event extraction. We were particularly interested in the results involving PRON and DNP types, since the participants of events resulting from resolving these types can potentially span multiple sentences, playing a role in our higher level goal of discourse interpretation. We manually analyzed the events extracted through resolution of PRON and DNP types of coreference. We found that 32.5% of such events were correct, however the positive effect was largely limited to intra-sentential coreference resolution (43.2% vs. 16%). Among the events correctly identified due to intra-sentential coreference resolution, 56% involved coreference of PRON type. On the other hand, among those due to inter-sentential coreference resolution, 84% involved the DNP type. In the following example, the possessive adjective *their *(PRON type) refers to the proteins *GATA3 *and *FOXP3 *and we extract the relevant events shown in (23b).

(23)   (a) *Thus, although GATA3 and FOXP3 showed similar kinetics, their**expression **polarizes at the end ...*

(b) *expression:*GENE_EXPRESSION(*e*_1_*, t*_1_) ^ *GATA3:*PROTEIN*(t*_1_*)*

*expression:*GENE_EXPRESSION(*e*_2_*, t*_2_) ^ *FOXP3:*PROTEIN*(t*_2_*)*

In Example (24), we correctly identify the event in (24b) from the sentence in (24a) by resolving the inter-sentential coreference between *this restriction factor *and *APOBEC3G*:

(24)   (a) *APOBEC3G (A3G), a member of the recently discovered family of human cytidine deaminases, is expressed in peripheral blood lymphocytes and has been shown to be active against HIV-1 and other retroviruses. To gain new insights into the **transcriptional regulation **of this restriction factor, ...*

(b) *transcriptional*_*regulation:*REGULATION(*e*_1_*,t*_1_) ^ *APOBEC3G:*PROTEIN*(t*_1_*)*

Among the misidentified events, we observe that some are due to shortcomings of the event extraction algorithm, rather than coreference resolution. In the following example, the coreference between the expression *these receptors *and the entities *CD3, CD2, and CD28 *is correctly identified; however, we extract the event annotation in (25b), since we ignore the quantifier *any*. The gold standard annotations are as given in (25c).

(25)   (a) *CD3, CD2, and CD28 are functionally distinct receptors on T lymphocytes. **Engagement **of any of these receptors induces the rapid tyrosine phosphorylation of a shared group of intracellular signaling proteins, ...*

(b) *engagement:*BINDING(*e*_1_*, t*_1_*,t*_2_) ^ *CD2:*PROTEIN*(t*_1_*) *^ *CD28:*PROTEIN*(t*_2_*)*

(c) *engagement:*BINDING(*e*_1_*, t*_1_) ^ *CD2:*PROTEIN*(t*_1_*)*

*engagement:*BINDING(*e*_2_*, t*_2_) ^ *CD28:*PROTEIN*(t*_2_*)*

We also noted cases in which the events that our system identifies due to coreference resolution seem correct, even though they are not annotated as such in the gold standard, as exemplified below. In this example, the anaphoric expression *their *is found to corefer with *IL-2 and IFN-γ*, and therefore, the event annotations in (26b) are extracted, whereas the gold standard only includes the event annotation in (26c).

(26)   (a) *Runx1 activates IL-2 and IFN-γ gene expression in conventional CD4+ T cells by **binding **to their respective promoter ...*

(b) *binding:*BINDING(*e*_1_*, t*_1_*,t*_2_) ^ *Runx1:*PROTEIN*(t*_1_*) *^ *IL-2:*PROTEIN*(t*_2_*)*

*binding:*BINDING(*e*_2_*, t*_1_*,t*_3_) ^ *Runx1:*PROTEIN*(t*_1_*) *^ *IFN-γ:*PROTEIN*(t*_3_*)*

(c) *binding:*BINDING(*e*_1_*, t*_1_) ^ *Runx1:*PROTEIN*(t*_1_*)*

However, the shortcomings of the coreference resolution are evident in most error cases. The fact that we only consider semantically bound elements as potential antecedents leads to a considerable number of errors. In such cases, the actual antecedent closer to the anaphoric expression may be ignored, in favor of a more distant entity. In the following example, we identify as antecedent *PKD1, PKD2, and PKD3 *for the pronoun *they*, because the actual antecedent, *PKD enzymes*, is semantically free. This leads to three false positive errors shown in (27b).

(27)   (a) *The protein kinase D (PKD) serine/threonine kinase family has three members: PKD1, PKD2, and PKD3. Most cell types express at least two PKD isoforms but PKD enzymes are especially highly expressed in haematopoietic cells, where they are activated in response to antigen receptors stimulation*.

(b) *activated:*POSITIVE_REGULATION(*e*_1_*, t*_1_) ^ *PKD1:*PROTEIN*(t*_1_*)*

*activated:*POSITIVE_REGULATION(*e*_2_*, t*_2_) ^ *PKD2:*PROTEIN*(t*_2_*)*

*activated:*POSITIVE_REGULATION(*e*_3_*, t*_3_) ^ *PKD3:*PROTEIN*(t*_3_*)*

## Conclusions and future work

Our two-phase, compositional approach to event extraction clearly distinguishes general linguistic principles from task-specific aspects. Our results demonstrate the viability of our approach on both abstracts and article bodies. The fact that we perform similarly on abstracts and article bodies is a particularly important aspect of our system. Our system also performs consistently between test sets and development sets, suggesting that it is robust and does not suffer from the brittleness and low recall often attributed to rule-based systems. We consider this robustness a result of the generality of the underlying rules, partially aided by syntactic dependency parsing as it normalizes much of the syntactic variation. The results also reveal some of the shortcomings of our approach. For example, our error analysis shows that some aspects of our semantic composition algorithm (argument propagation, in particular) requires more refinement. We also find that learning trigger expressions for the common event types in ID and GENIA tracks from both training corpora has a negative effect on the ID track results; however, more research is needed to determine whether GENIA and ID texts really constitute two different sublanguages or whether the differences are simply due to annotation inconsistencies.

While biological event extraction at the sentence level is already a challenging task, we believe that future research should also focus on moving beyond sentence level to wider discourse context. An important step in this direction is coreference resolution, a problem that we investigated post-shared task. We did not observe much significant improvement due to coreference resolution; however, our experiments allowed us to identify several areas of improvement. For example, the underspecified nature of our current coreference resolution algorithm (that it only targets PROTEIN and predicate terms as antecedents) leads us to miss some relatively easy cases of PRON and DNP types of coreference and lowers precision. Integrating a named-entity recognizer (NER) into our system would allow us to impose more semantics on our system, and thus, could improve coreference resolution performance. We expect that a general NER system such as MetaMap [46] which provides access to the rich semantics of UMLS [47] would be particularly useful. In addition, coreference resolution interacts with higher level discourse constraints in significant ways (see, for example, [48]), and we are currently exploring this further. Our modular, incremental approach ensures that new capabilities can be added and their effect on overall system performance can be measured. With these improvements, we plan to make our system available to the scientific community as a robust baseline system in the near future.

## Competing interests

The authors declare that they have no competing interests.

## Authors' contributions

HK conceived of the study, developed the system, performed the analyses, and drafted the manuscript. SB directed the research and helped draft the manuscript. All authors read and approved the final manuscript.
